# The Effect of Oral Probiotics (Streptococcus Salivarius k12) on the Salivary Level of Secretory Immunoglobulin A, Salivation Rate, and Oral Biofilm: A Pilot Randomized Clinical Trial

**DOI:** 10.3390/nu14051124

**Published:** 2022-03-07

**Authors:** Ksenia Babina, Dilara Salikhova, Maria Polyakova, Oxana Svitich, Roman Samoylikov, Samya Ahmad El-Abed, Alexandr Zaytsev, Nina Novozhilova

**Affiliations:** 1 Department of Therapeutic Dentistry, I.M. Sechenov First Moscow State Medical University (Sechenov University), 119991 Moscow, Russia; salikhova_d_i@student.sechenov.ru (D.S.); polyakova_m_a_1@staff.sechenov.ru (M.P.); novozhilova_n_e@staff.sechenov.ru (N.N.); 2 Department of Microbiology, Virology, and Immunology, I.M. Sechenov First Moscow State Medical University (Sechenov University), 119991 Moscow, Russia; svitich_o_a@staff.sechenov.ru; 3 I.I. Mechnikov Research Institute of Vaccines and Sera, 105064 Moscow, Russia; roma_sam78@mail.ru (R.S.); sam9520177@yandex.ru (S.A.E.-A.); 4 Institute of Linguistics and Intercultural Communication, I.M. Sechenov First Moscow State Medical University (Sechenov University), 119991 Moscow, Russia; zaytsev_a_b@staff.sechenov.ru

**Keywords:** probiotics, *Streptococcus salivarius* K12, dental biofilm, salivary secretory immunoglobulins A, unstimulated salivary flow rate

## Abstract

We aimed to assess the effect of oral probiotics containing the *Streptococcus salivarius* K12 strain on the salivary level of secretory immunoglobulin A, salivation rate, and oral biofilm. Thirty-one consenting patients meeting the inclusion criteria were recruited in this double-blind, placebo-controlled, two-arm, parallel-group study and randomly divided into probiotic (*n* = 15) and placebo (*n* = 16) groups. Unstimulated salivation rate, concentration of salivary secretory immunoglobulin A, Turesky index, and Papillary-Marginal-Attached index were assessed after 4 weeks of intervention and 2 weeks of washout. Thirty patients completed the entire study protocol. We found no increase in salivary secretory immunoglobulin A levels and salivary flow rates in the probiotic group compared with placebo. Baseline and outcome salivary secretory immunoglobulin A concentrations (mg/L) were 226 ± 130 and 200 ± 113 for the probiotic group and 205 ± 92 and 191 ± 97 for the placebo group, respectively. A significant decrease in plaque accumulation was observed in the probiotic group at 4 and 6 weeks. Within the limitations of the present study, it may be concluded that probiotic intake (*Streptococcus salivarius* K12) does not affect salivation rates and secretory immunoglobulin A salivary levels but exhibits a positive effect on plaque accumulation. Trial registration NCT05039320. Funding: none.

## 1. Introduction

The oral cavity harbors the second largest microbiome in the human body. This microbial community, hosting over 700 species [1], is based on the interactions of microorganisms with the host environment as well as on their interaction with each other [2]. A healthy oral cavity is characterized by a dynamic balance between commensal (i.e., non-infectious) and opportunistic (cariogenic) microorganisms. This equilibrium can be disturbed by a high-carbohydrate diet, poor oral hygiene, some medications, and systemic diseases [3]. Dental caries, the most common non-communicable disease worldwide [4], are primarily caused by an imbalance in the oral microbiome, i.e., a predominance of cariogenic microorganisms, including various types of *streptococci* and *lactobacilli*, *actinomyces*, *bacteroides*, and *bifidobacteria* [5]. In this regard, the replacement of the cariogenic microorganisms with commensals is one of the possible strategies to prevent dental caries. This can be achieved by using drugs (supplements) that restore the balance of microflora, i.e., oral biotics and probiotics [6].

According to the WHO, probiotics are live microorganisms that, when administered in adequate amounts, confer a health benefit on the host [7]. The idea that ingestion of certain microorganisms could be beneficial to the gastrointestinal tract was first suggested by the Russian Nobel laureate Élie Metchnikoff [8]. Since then, a number of studies have proven the effectiveness of probiotics for the prevention and treatment of gastrointestinal [9], allergic [10], and respiratory [11,12] diseases.

A study by Miller et al. was one of the first to investigate the use of various microorganisms for dental purposes [13]. Later, some studies proved a decrease in the number of oral pathogens caused by probiotic intake. In particular, many studies have shown decreased counts of *Streptococcus mutans* and *Candida albicans* [6,14,15,16,17]. The oral microflora in this case is supposed to become more heterogeneous with a predominance of the commensals. It is assumed that probiotic bacteria compete with cariogenic microorganisms for adhesion sites or food substrates and affect immune mechanisms through secretion of antimicrobial substances [18]. Therefore, taking probiotics can lead not only to a reduction in certain pathogens but also to transformations of the entire oral microbiome composition [19,20].

In addition to the presence of cariogenic microorganisms and nutrient substrates (high-carbohydrate foods), caries development requires sufficient contact time between the microorganism and susceptible tissues [21]. Probiotics can also reduce caries risk by increasing salivary flow rate [22] and hence reducing the time that microorganisms are in contact with the tooth surface.

In addition, researchers have found that administration of probiotics may increase secretory immunoglobulin A (sIgA) levels in saliva [23,24,25]. Immunoglobulins inhibit adherence of microorganisms, protect the host against absorption of antigens from mucosal surfaces, inhibit inflammatory effects, enhance phagocytosis, and neutralize microbial toxins and invasive pathogens [26]. Secretory immunoglobulin A is one of the major salivary immunoglobulins that plays an important role in caries prevention [27]. It may be hypothesized that due to the transformation of the oral microbiome, increase in salivary flow rate, and increase in secretion of sIgA probiotics can reduce the rate of plaque formation [15].

In dentistry, the most commonly used probiotics are those containing lacto- and bifidobacteria, including Lactobacillus casei, Lactobacillus paracasei, Lactobacillus acidophilus, Lactobacillus rhamnosus, Lactobacillus gasseri, Lactobacillus reuteri, Bifidobacterium bifidum, Bifidobacterium infantis, and Bifidobacterium subtilis [28]. However, these strains are often unsuccessful in colonizing oral tissues, which is why a new generation of probiotic strains containing streptococci has recently been developed.

Among the groups of streptococci present in the oral microbiome are *mitis*, *sanguinis*, *anginosus*, *salivarius*, *downei*, *mutans*, *pyogenic*, and *bovis* [29]. *Streptococcus salivarius* is of particular importance and is the subject of extensive research, as this microorganism plays an important role in regulating the balance within the microbial communities of the gastrointestinal tract [30]. It is one of the first bacteria to colonize oral mucosa in the first few days after birth [31]. This important commensal has been demonstrated to inhibit the growth of the important cariogenic *streptococci* (*Streptococcus mutans* and *Streptococcus sobrinus*) by competing for tooth sites during initial oral colonization [32,33]. *S. salivarius* was also isolated from the dental plaque biofilm of a caries-free child with healthy oral tissues [34]. Miller et al. demonstrated a reduction in plaque formation when *S. mutans*, the main cariogenic microorganism, was co-cultured with *S. salivarius*, *Streptococcus faecalis*, or *L. casei* compared to pure cultures of *S. mutans* [13]. *S. salivarius* produces bacteriocins, inhibits colonization of the epithelium by *Aggregatibacter actinomycetemcomitans*, and protects against *C. albicans* invasion by inhibiting adhesion through mechanisms independent of its antimicrobial activity [33]. This microorganism has also been demonstrated to affect the immune response by inhibiting the inflammatory pathways activated by different pathogens [33]. Due to its properties, *S. salivarius* can be successfully used to prevent and treat ENT (ear, nose and throat) inflammatory diseases [35], halitosis [36], candidiasis [37], and dental caries [38,39,40]. Among the *S. salivarius*-containing probiotics, the two most promising strains are K12 and M18 [31].

Despite a large number of studies, there is still no consensus on the ways in which probiotics affect dental health indicators. Moreover, there is a paucity of literature on the use of *S. salivarius* (particularly K12 strain) as a probiotic for dental purposes. The aim of our study is to assess the effect of oral probiotics containing *Streptococcus salivarius* K12 strain on the salivary level of secretory immunoglobulin A (sIgA), salivation rate, and oral biofilm.

## 2. Materials and Methods

### 2.1. Ethical Approval

This clinical study was approved by the Local Ethics Committee (Protocol no. 34-20 (9 December 2020)) and registered on clinicaltrials.gov registry NCT05039320). This research received no external funding. The trial was designed following the principles of the modified Helsinki’s code for human clinical studies (2013) and the CONSORT 2010 guidelines for reporting randomized clinical trials.

### 2.2. Study Design

The study assessed the effect of oral probiotics containing *Streptococcus salivarius* K12 on oral biofilm, salivation rate, and secretory immunoglobulin A (sIgA) salivary level. A double-blind, randomized, two-arm parallel-group study was conducted from September 2021 to November 2021.

General visit descriptions and study schedule are presented in Table 1.

### 2.3. Sampling Criteria

The patients visiting the Dental Institute were invited to participate in the study. Thirty-one healthy adult volunteers aged 20–24 years were enrolled and assigned to interventions by one of the study authors (DS). Written informed consent was obtained from all patients for participation in the study and publication of the data for research and education purposes. The patients were recommended to brush their teeth using standardized technique (Bass) and pea-sized amount of toothpaste without any antibacterial or antiplaque components twice daily.

#### 2.3.1. Inclusion Criteria

- Permanent dentition;
- Presence of more than 20 teeth;
- Absence of systemic and chronic diseases.

#### 2.3.2. Exclusion Criteria

- More than 5 cavities requiring treatment;
- Refusal to sign informed consent;
- Taking supplements or lozenges containing probiotics or prebiotics 3 weeks before the study;
- Taking antibiotics (within 1 month before the study);
- Orthodontic and prosthetic treatment;
- Allergy to the components of the drugs used in the study;
- Use of other hygiene products, immunostimulants and antibacterials, probiotics, or prebiotics during the study;
- Refusal to take a given medication;
- Failure to attend check-ups.

### 2.4. Randomization

Subjects who met all inclusion and none of the exclusion criteria were randomized to one of the following study groups: Group 1 received lozenges containing a probiotic (*Streptococcus salivarius* K12); Group 2 received placebo lozenges. The allocation concealment was performed using containers numbered by a “third party” (person who did not participate in the study). The lozenges in unlabeled bottles were placed in the containers. The probiotics and placebo lozenges were identical in taste, color, texture, and size, but the placebo lozenges did not contain active bacteria. Each volunteer on enrolment received a container of lozenges. Neither study subjects nor researchers were aware of the type of lozenges used.

### 2.5. Interventions

All participants took probiotics/placebos for 4 weeks, 1 lozenge per day (Table 2). The intervention was followed by a two-week washout period, during which the prescribed lozenges were not taken. This period was introduced to assess the stability of the achieved results.

### 2.6. Outcomes

Primary outcome measures included concentration of secretory immunoglobulin A in saliva and unstimulated salivary flow rate. Secondary outcome measures included Turesky modification of the Quigley-Hein plaque index (TQHPI) and the papillary marginal attached index (PMA). The evaluations were performed at baseline and 4 and 6 weeks by a single operator (DS).

TQHPI, PMA, and DMFT (decayed, missing, and filled teeth index) were accessed as described elsewhere [41,42,43]. Salivary concentration of sIgA was determined with the help of ELISA and using the salivary secretory IgA indirect enzyme immunoassay kit (8668 IgA secretory ELISA-BEST kit, VectorBest, Novosibirsk, Russia), in accordance with the manufacturer’s instructions.

For unstimulated salivary flow measurements, participants refrained from eating, drinking, smoking, or conducting oral hygiene procedures for a minimum of 90 min prior to salivary collection. To avoid diurnal variations in saliva output, all measurements were taken in the morning. Participants were comfortably seated and, after a few minutes of relaxation, were trained to avoid swallowing saliva and asked to lean forward and spit all the saliva they produced every 2 min through a glass funnel and into a graduated test tube. The volume of the saliva collected over the 10-min period was measured. The flow rate was determined according to the following formula: Salivation rate (ml/min) = saliva volume (mL)/saliva collection time (min).

### 2.7. Statistical Analysis

The sample size for this pilot study was defined based on the sample sizes of similar studies [44,45,46]. All analyses were performed using per-protocol population. We analyzed all subjects who did not substantially deviate from the protocol as to be determined on a per-subject basis by the study’s principal investigator (KB) immediately before database lock.

Data were presented as means and standard deviations with 95% confidence intervals, medians and 25 and 75 percentiles, and percentages depending on the type of variables. The normality and sphericity of distribution of continuous variables were assessed with Shapiro–Wilk and Levene’s tests, respectively. If the assumptions of normality and sphericity were met, repeated measures mixed ANOVA was performed followed by the post hoc Tukey’s test with adjustment for multiple comparisons. If the aforementioned assumptions were not met, the differences between the groups were assessed using the Mann–Witney U-test and the differences within the groups at different study timepoints were assessed with Friedman test with post hoc comparisons. The same non-parametric tests were used for the analyses of categorical and ordinal variables. Fisher’s exact test was used to access the frequencies of categorical variables in the groups.

### 2.8. Data Management

Data entry was completed in the RedCap database. The data were exported into CSV file format, which was then used for data analysis (only de-identified data) in R version 3.6.0 (26 April 2019), using the following packages: “doBy,” “rstatix,” “tidyverse,” “ggpubr,” “stats,” “effectsize,” “psych,” “PMCMRplus,” “lawstat,” “library,” “corrplot,” and “Hmisc.”

## 3. Results

The study sample included 31 volunteers (27 females and 3 males) aged 20–24 years (mean: 21.2 ± 0.8 years). They were divided into the placebo group (14 females and 2 males) and the probiotic group (14 females and 1 male) using a random sequence generator. There were no significant differences between the groups in age, gender distribution, DMFT, and the decay component of DMFT values (Table 3).

**Figure 1:**
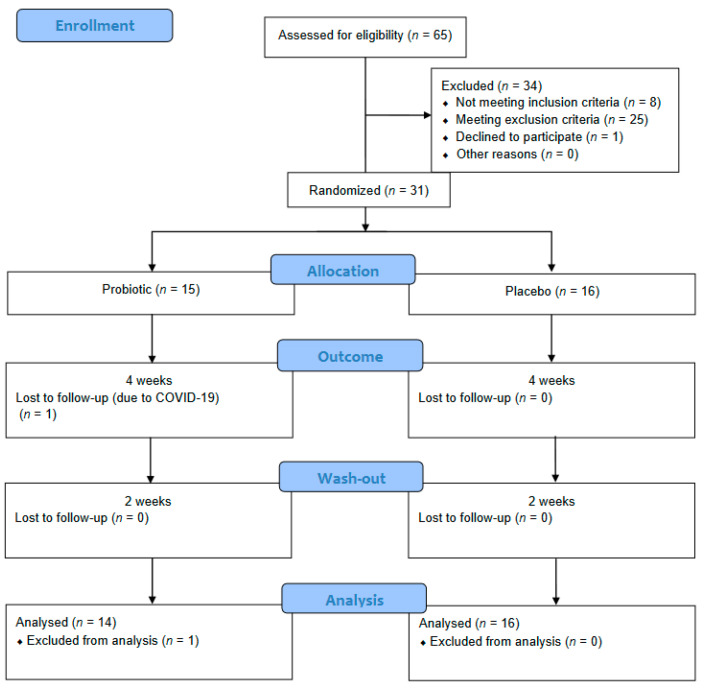
Patient flow diagram.

Figure 1 shows the patient flow diagram. Of the 31 individuals included in this study, 30 completed the entire study protocol. One participant from the probiotic group was lost to follow-up due to sickness not related to the intervention (COVID-19). No adverse effects were registered.

The baseline, outcome, and washout values of salivary IgA, unstimulated salivary flow rate, and dental indices are presented in Table 4. We found no statistically significant differences in the salivary secretory immunoglobulin A (sIgA) levels and unstimulated salivary flow rates between the individuals who took probiotics and placebos. After a 4-week probiotic intervention and a 2-week washout period, the study participants had significantly lower TQHPI values than the controls. At baseline, seven participants (three in the probiotic group and four in the control group) had PMA index values greater than 0. The PMA values tended to decrease in the probiotic group at the outcome and washout timepoints, although these changes did not reach the level of statistical significance (*p* = 0.06081).

There were no statistically significant differences in the distribution of the study subjects across the levels of salivary sIgA and PMA in the probiotic and placebo groups at all study timepoints (Table 5).

**Figure 2:**
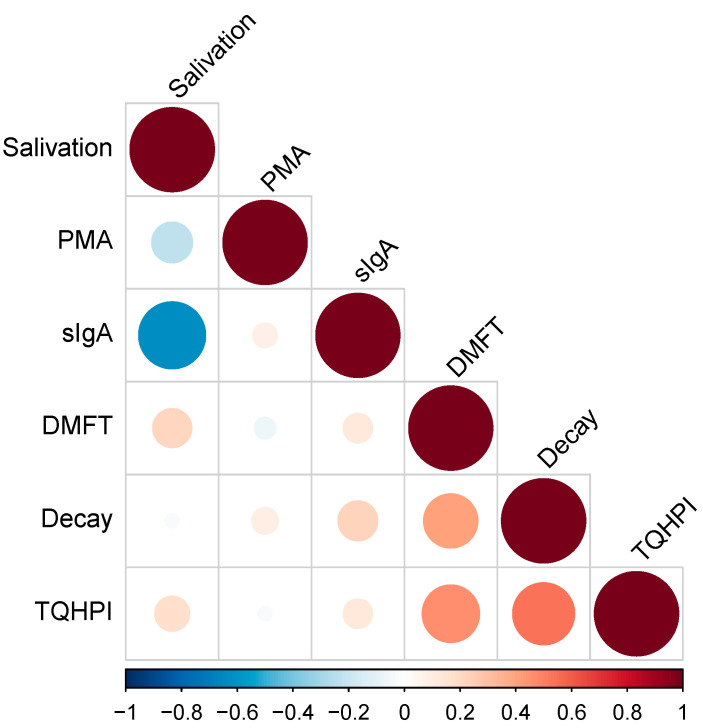
Correlation analysis. TQHPI—Turesky modification of the Quigley-Hein plaque index; DMFT—decayed, missing, and filled teeth index; sIgA—secretory immunoglobulin A; PMA—papillary marginal attached index.

Figure 2 shows the results of the correlation analysis of oral health indicators. We found a strong negative correlation between salivation rate and sIgA level (r = −0.62), *p* = 0.000239395). A moderate positive correlation was detected between the number of decayed teeth and TQHPI values (r = 0.57, *p* = 0.000968335). No significant correlation was observed between the number of the decay component of DMFT and sIgA level (r = 0.17, *p* = 0.379).

## 4. Discussion

In the present study, we assessed the effect of oral probiotics containing *Streptococcus salivarius* K12 on secretory immunoglobulin A salivary levels, salivation rates, and oral biofilm in healthy adults. We found no increase in salivary secretory immunoglobulin A (sIgA) levels and salivary flow rates in the probiotic group compared with the placebo group. However, we observed a significant decrease in plaque accumulation in the probiotic group after 2 and 4 weeks of probiotic intake. A decrease in the PMA index was observed in the probiotic group, although the differences did not reach the level of statistical significance. This was possibly due to a small number of patients with gingivitis.

Salivary IgA is an important protein that participates in the prevention of oral diseases. sIgA level determination is widely used in dental science, as it can be collected noninvasively and is considered an indicator of health and disease [47]. Probiotics demonstrated beneficial effect on host immune response [48], although studies of the effect of probiotics on sIgA levels have shown conflicting results. Some studies found increased levels of salivary sIgA in adults [44,47,49,50,51], elderly patients [52], and children [47,48] after probiotic intake, while others were unable to confirm such findings [23,53,54,55,56]. One study even demonstrated a decrease in salivary sIgA after Bifidobacterium-containing probiotic intake [57]. A meta-analysis by Ebrahimpour et al. demonstrated no significant effect of probiotic intake on salivary sIgA levels compared to placebo [58], which is in line with our results. In the present study, the analysis of variance showed that neither the time factor (baseline/outcome/washout) nor the group factor (probiotic/placebo) affected the salivary sIgA levels.

Salivary flow rate is another crucial parameter for the maintenance of oral and systemic health [59]. There is some evidence that probiotics may affect salivary flow rate [59,60]. However, other studies did not confirm this effect of probiotics [61,62,63,64]. Our results are in agreement with the latter studies: we found no increase in unstimulated salivary flow rate after the intake of probiotics compared with placebo.

Differences in the effects of probiotics on various health indicators may be explained by the age of participants. In the studies involving elderly people, antibody responses might be different from healthy adults [58]. Moreover, intra- and inter-individual variations in saliva volume and its contents are influenced by a variety of factors, such as cigarette smoking [47,54], chronic and acute stress [47,54,58], depression [47,54], and circadian variation [47,65].

Immune-modulatory effects of probiotics in general and for particular species may be strain-specific [54]. To the best of our knowledge, there were no published reports directly comparable to ours. The only report partially comparable to ours was that of Ferrer et al., who assessed the effect of topical application of *Streptococcus*-containing probiotics on plaque accumulation, saliva quality, and salivary flow [22]. They found a significant increase in salivary flow rate at day 15 in the probiotic group compared with the placebo group. Furthermore, in the probiotic group, there was a decrease in the amount of dental plaque and gingival inflammation, but no differences were observed in the placebo group [22]. A similar effect on plaque formation was reported by Burton at al., who demonstrated that the probiotic strain *S. salivarius* M18 administered twice daily caused a significant reduction in plaque formation in children [66]. In the present study, we observed a significant decrease in TQHPI in the probiotic group after 2 and 4 weeks of probiotic intake.

However, the plaque-reducing effect of probiotics may also be strain-specific and product-specific. According to a meta-analysis by Nadelman et al., dairy probiotics increased plaque accumulation, possibly due to an increase in the amount of carbohydrates [67].

It could be expected that a decrease in plaque index would result in a decrease in gingivitis (PMA score). A number of studies have demonstrated that probiotics significantly improved various gingival health indicators, i.e., decreased gingival indices and bleeding on probing [22,42,68,69]. In our study, a decrease in the PMA index was observed in the probiotic group, although the differences did not reach the level of statistical significance. This was possibly due to a small number of patients with gingivitis due to good or moderate levels of oral hygiene in the majority of patients in both groups. Similarly, Montero et al. reported insignificant changes in the mean gingival index in general, although it significantly reduced at the sites of severe inflammation [70].

Although plaque accumulation rate is a rapidly changing variable and caries development is a relatively slow process, we found a moderate positive correlation between TQHPI values and the number of decayed teeth. There was no significant correlation between the value of the decay component of DMFT and sIgA level, although some authors hypothesized that the level of salivary sIgA may serve as a predictor of caries resistance in a patient [27,71,72,73].

According to the literature, protein concentrations in saliva may also be dependent on the changes in salivary flow rates [74,75], so they may be sIgA levels [76,77]. For example, an increase in salivary sIgA in people with xerostomia was reported in pregnant women [78] and students experiencing stress because of exams [74]. We found a strong negative correlation between salivation rates and sIgA levels (*p* < 0.001). Similarly, an inverse correlation between salivary flow rates and salivary sIgA concentrations has been reported in previous studies [79,80,81,82,83].

We readily acknowledge several limitations to our study. This was a pilot study; the relatively small number of participants was defined based on the sample sizes of similar studies [44,45,46]. Further research with a larger sample size is planned based on the data generated in the present study. A four-week intake of probiotics and two-week washout period are relatively short periods for the assessment of the effect of probiotics; however, similar timepoints were used in the previous studies [47,54,84]. Moreover, although probiotics may affect different salivary components [25,58,63,85], we assessed the influence of probiotics on a single salivary protein (sIgA), having hypothesized that this parameter would be the most sensitive one to probiotic intake.

## 5. Conclusions

Within the limitations of this pilot study, it can be concluded that probiotic intake (*Streptococcus salivarius* K12) does not affect salivation rates and secretory immunoglobulin A salivary levels in healthy adults. However, a decrease in plaque accumulation was observed.

## Figures and Tables

**Table 1:** Study schedule.

	Screening Visit	Baseline Visit	4 Weeks	6 Weeks
Eligibility assessment:				
Inclusion	X			
Exclusion	X	X	X	X
DMFT	X	X	X	X
TQHPI	X	X	X	X
Oral hygiene instructions	X			
PMA		X	X	X
Unstimulated salivary flow rate		X	X	X
Salivary sIgA		X	X	X

DMFT—Decayed, missing, and filled teeth index; TQHPI—Turesky modification of the Quigley-Hein plaque index; PMA—papillary marginal attached index; sIgA—secretory immunoglobulin A.

**Table 2:** Arms’ characteristics.

Group	Dietary Supplement Composition	Intervention
Group 1—probiotic (“Bactoblis”)	*Streptococcus salivarius* K12 (≥1 × 10^9^ CFU in 1 tablet), fructose (sweetener), maltodextrin, silicon dioxide, magnesium stearate (vegetable), flavoring (strawberry)	Dissolve the lozenges in the mouth once a day for 4 weeks
Group 2—placebo	Fructose (sweetener), maltodextrin, silicon dioxide, magnesium stearate (vegetable), flavoring (strawberry)	

CFU—Colony-forming unit.

**Table 3:** Characteristics of subjects at inclusion.

Parameter	Total (*n* = 30)	Probiotics (*n* = 14)	Placebo (*n* = 16)	Statistical Significance
Sex, n (%)				
Female	27 (90)	13 (93)	14 (87.5)	*p*-value = 1.0 ^a^
Male	3 (10)	1 (7)	2 (12.5)	
Age				
m (sd)	21.2 (0.8)	21.4 (0.9)	20.9 (0.6)	*p*-value = 0.171 ^b^
Median [Q1; Q3]	21 [21; 22]	21 [21; 22]	21 [20.75; 21]	
min-max	20–24	21–24	20–22	
DMFT				
Median [Q1; Q3]	9 [6.25; 12.5]	9 [7.5; 10.75]	10.5 [5.75; 14]	*p-*value = 0.4892 ^b^
min-max	0–20	0–20	2–17	
Decay				
Median [Q1; Q3]	2.5 [2; 4]	3.5 [2; 4]	2 [2; 3]	*p-*value = 0.3389 ^b^
min-max	0–5	0–5	0–5	

^a^ Fisher’s exact test; ^b^ Mann–Whitney–Wilcoxon test; DMFT—decayed, missing, and filled Teeth index.

**Table 4:** Summary of evaluated parameters.

Parameter	Probiotics (*n* = 14)	Placebo (*n* = 16)	Statistical Analysis
sIgA, mg/L, m (sd)			
Baseline	226 (130)	205 (92)	Arm: F = 0.385, *p-*value = 0.54
Outcome	200 (113)	191 (97)	Time: F = 0.572, *p-*value = 0.568
Washout	227 (119)	196 (114)	Arm*Time: F = 0.16, *p-*value = 0.853 ^a^
Salivation, mL/min, m (sd)			
Baseline	0.47 (0.20)	0.48 (0.18)	Arm: F = 0.002, *p-*value = 0.969
Outcome	0.55 (0.25)	0.53 (0.17)	Time: F = 2.952, *p-*value = 0.060
Washout	0.53 (0.22)	0.53 (0.13)	Arm*Time: F = 0.234, *p-*value = 0.792 ^a^
TQHPI, median [Q1; Q3]			
Baseline	2.8 [2.5; 3.1]	2.9 [2.7; 3.1]	*p-*value = 0.5744 ^b^
Outcome	2.5 [2.2; 2.9]	2.9 [2.8; 3.2]	*p-*value = 0.01114 ^b^
Washout	2.5 [2.3; 2.8]	3.0 [2.9; 3.3]	*p-*value = 0.009286 ^b^
Within-group comparisons	*p-*value = 0.02437 ^c^	*p-*value = 0.1642 ^c^	
PMA > 0, units, median [Q1; Q3]	*n* = 3	*n* = 4	
Baseline	4 [3; 6.5]	2.5 [2; 3.2]	*p-*value = 0.4587 ^b^
Outcome	0 [0; 1.5]	3.5 [2.2; 4.2]	*p-*value = 0.2664 ^b^
Washout	0 [0; 0]	3.5 [2.2; 4.2]	*p-*value = 0.1187 ^b^
Within-group comparisons	*p-*value = 0.06081 ^c^	*p-*value = 0.5836 ^c^	

^a^ Mixed ANOVA model; ^b^ Mann–Witney–Wilcoxon test; ^c^ Friedman rank sum test; sIgA—secretory immunoglobulin A; TQHPI—Turesky modification of the Quigley-Hein plaque index; PMA—papillary marginal attached index.

**Table 5:** Distribution of sIgA levels (low, normal, high) ^a^ and PMA (zero or greater than zero) in the study groups.

	Probiotics (*n* = 14)	Placebo (*n* = 16)	Significance ^b^
Level of sIgA			
Baseline, *n* (%)			
Low	3 (21.5)	4 (25.0)	*p-*value = 0.883
Normal	8 (57.0)	10 (62.5)	
High	3 (21.5)	2 (12.5)	
Outcome, *n* (%)			
Low	4 (28.5)	5 (31)	*p-*value = 0.8959
Normal	6 (43.0)	8 (50)	
High	4 (28.5)	3 (19)	
Washout, *n* (%)			
Low	3 (21.5)	2 (12.5)	*p-*value = 0.4369
Normal	7 (50.0)	12 (75.0)	
High	4 (28.5)	2 (12.5)	
PMA			
Baseline, *n* (%)			
PMA = 0	11 (79)	12 (75)	*p-*value = 1.0
PMA > 0	3 (21)	4 (25)	
Outcome, *n* (%)			
PMA = 0	13 (93)	13 (81)	*p-*value = 0.6015
PMA > 0	1 (7)	3 (19)	
Washout, *n* (%)			
PMA = 0	14 (100)	13 (81)	*p-*value = 0.2276
PMA > 0	-	3 (19)	

^a^ According to manufacturer’s instructions (IgA secretory ELISA-BEST kit); ^b^ Fisher’s exact test; sIgA—secretory immunoglobulin A; PMA—papillary marginal attached index.

## Data Availability

The datasets used and/or analyzed during the current study are available from the corresponding author upon reasonable request.
